# Maternal elevated salt consumption and the development of autism spectrum disorder in the offspring

**DOI:** 10.1186/s12974-019-1666-2

**Published:** 2019-12-14

**Authors:** Kazi Farhana Afroz, Karina Alviña

**Affiliations:** 1 Department of Biological Sciences, Texas Tech University, 2901 Main St. Room #05, Biology Building, Lubbock, TX 79409 USA; 2 Department of Neuroscience, University of Florida, 1149 Newell Drive, Room L1-100, Gainesville, FL 32611 USA

**Keywords:** Autism spectrum disorder, High salt, Autoimmunity, Gut microbiome, T helper cells, Interleukin-17

## Abstract

Autism spectrum disorder (ASD) is a prevalent neurodevelopmental condition with no known etiology or cure. Several possible contributing factors, both genetic and environmental, are being actively investigated. Amongst these, maternal immune dysregulation has been identified as potentially involved in promoting ASD in the offspring. Indeed, ASD-like behaviors have been observed in studies using the maternal immune activation mouse model. Furthermore, recent studies have shed light on maternal dietary habits and their impact on the gut microbiome as factors possibly facilitating ASD. However, most of these studies have been limited to the effects of high fat and/or high sugar. More recent data, however, have shown that elevated salt consumption has a significant effect on the immune system and gut microbiome, often resulting in gut dysbiosis and induction of pro-inflammatory pathways. Specifically, high salt alters the gut microbiome and induces the differentiation of T helper-17 cells that produce pro-inflammatory cytokines such as interleukin-17 and interleukin-23. Moreover, elevated salt can also reduce the differentiation of regulatory T cells that help maintaining a balanced immune system. While in the innate immune system, high salt can cause over activation of M1 pro-inflammatory macrophages and downregulation of M2 regulatory macrophages. These changes to the immune system are alarming because excessive consumption of salt is a documented worldwide problem. Thus, in this review, we discuss recent findings on high salt intake, gut microbiome, and immune system dysregulation while proposing a hypothesis to link maternal overconsumption of salt and children’s ASD.

## Introduction

According to the United States (US) Center for Disease Control and Prevention (CDC), autism spectrum disorder (ASD) is a group of neurodevelopmental disorders characterized by significant challenges in social and communication behaviors, usually appearing in early childhood and persisting throughout the individuals’ life [10]. ASD is typically recognized in children between 1 and 2 years of age, where differences in communication, social interaction, and social learning are first observed [55]. The WHO (World Health Organization) reports that currently 1 in 160 children worldwide are diagnosed with ASD [52]. Though this figure is likely an underestimation of the real prevalence of ASD, as in many developing countries, these statistics are not fully known. Nevertheless, based on the epidemiological studies conducted by the WHO for the last 50 years, it is concerning that the prevalence of ASD diagnosis has increased so rapidly. In 2017, approximately 1% of the world population was estimated to be affected by ASD [74].

Despite the growing number of ASD cases, the etiology of the disease is still unknown. The current consensus is that ASD is likely caused by the combination of genetic, environmental, and neurodevelopmental factors [44]. Early studies however focused on genetic predisposition. For instance, studies found that similarities between monozygotic twins reach 90% for ASD, as compared with less than 10% for dizygotic twins and siblings, and approximately 0.6–1.0% occurrence in the general population [1, 49]. Nevertheless, it quickly became evident that both environmental and epigenetic factors needed to be considered. Amongst these factors, the presence of altered gut microbiome and dysregulated immune system have been documented in ASD patients [4, 76, 94]. In fact, more recent studies have provided further evidence for the association between maternal immune dysregulation and children’s ASD. For example, induction of immune response by injecting lipopolysaccharide or synthetic virus in pregnant rodents resulted in ASD-like neurodevelopmental abnormalities in the offspring [17, 64]. Similarly, from human studies, it was reported that the risk of ASD in the offspring increased approximately 30% in mothers diagnosed with rheumatoid arthritis (RA), a common disease caused by dysregulation of the immune system [74].

As environmental factors, dietary habits such as high consumption of fat or salt (which can alter the maternal gut microbiome) have emerged as potentially related with ASD in the offspring [14]. Several recent studies have shown a correlation between high salt intake, gut dysbiosis, immune dysregulation, and cognitive dysfunction [9, 28, 45]. Although, a causal relation between parental high salt consumption and offspring ASD has not been fully demonstrated yet, there is substantial evidence to support this notion.

According to the WHO, much of the world population consumes more salt than the recommended amount [13, 69]. In fact, in the US alone, the average salt intake is almost twice the recommended amount [37]. It is a matter of concern as more than two thirds of the consumed salt come from foods that people eat in large quantities, such as bread, cured meats, canned foods, and fried foods [37]. Moreover, this diet rich in salt became a worldwide well-established cause of morbidity and mortality due its ability to cause hypertension, cardiovascular diseases, and kidney failure [81]. In addition, high salt diet can also drive an imbalance in the immune system, by inducing reactive T helper-17 (Th17) cells [45]. A recent study reported that consumption of elevated dietary salt not only induces the proliferation of pro-inflammatory immune cells, but also decreases the functional ability of regulatory immune cells to suppress it [9]. Further studies indicated that this Th17 axis dysregulation caused by high salt diet happens via gut dysbiosis [96]. Additionally, the imbalance in the immune system due to high salt diet was shown to promote cognitive dysfunction in mice, via a nitric oxide (NO)-dependent mechanism [28]. Specifically, high salt diet increased Th17 cell proliferation in the small intestine of mice, leading to an increase in the plasma level of a pro-inflammatory interleukin (IL)-17. High levels of IL-17 suppressed the production of the endothelial vasodilator NO. Decreased NO production resulted in reduced cerebral blood flow which contributes to cognitive impairment. These specific findings support the idea that high salt diet can influence the alteration of gut microbial composition and ultimately cause host-immune dysregulation [28].

In summary, several lines of evidence support a contributing relationship between maternal elevated salt consumption and offspring ASD. In this review, we discuss these recent findings and put forward a novel hypothesis that links dietary choices, specifically elevated salt, with the alarming increase in ASD diagnosis.

## Gut dysbiosis and immune system dysregulation in ASD patients

Humans and other animal species (including insects) are the hosts of a range of microorganisms. The skin, intestines, reproductive organs, and nasal and oral cavity of the human body are colonized by trillions of microorganisms that can survive in extreme condition and share a commensal relationship with the host [41, 67]. Among all organs, the intestines, also known as gut, are the biggest reservoir of these commensal microorganisms, which are collectively called the gut microbiome [6, 48]. Though the gut microbiome includes archaea, fungi, protozoa, helminths, and viruses, it is dominated by almost 2000 species of bacteria [62, 79]. Studies show that a healthy human between 20 and 30 years of age and weighing 70 kg (154 lb) carries around 39 trillion of bacterial cells while the human cell count is around 30 trillion [80].

Increasing evidence shows that the gut microbiome changes over the individual’s lifetime [93, 100]. The newborn microbiota for instance is low in diversity and dominated by two major phyla: *Proteobacteria* and *Actinobacteria* [73]. By the time the individual reaches adulthood, the microbiota becomes diverse with the dominance of phyla *Firmicutes* and *Bacteroidetes* [73]. Moreover, the adult microbiome is so distinct between different people that it could be seen as an alternative fingerprint [70, 88]. More recent studies have identified that a healthy adult human gut microbiota population is mostly comprised of three enterotypes (i.e., bacteriological classification based on gut microbiota ecosystem), namely, *Prevotella, Ruminococcus,* and *Bacteroides* [73, 100]. Based on this defined gut microorganism population any pathological change is called gut dysbiosis [66, 67].

Though it was previously assumed that most of the gut microflora colonization happens within the first 2 years after birth by the influence of surrounding environment, recent studies show that the gut microflora of a newborn child is very similar to the mother’s [93]. The presence of maternal bacterial DNA in the amniotic fluid, placenta, meconium, and fetal membranes supports the notion that before and right after birth, the child’s gut microbiota is mostly dominated by maternal microbes which later changes due exposure to diverse environmental conditions [86, 91]. Additionally, recent findings have shown that breast milk contains several microbes that can be very influential on the offspring’s gut and overall health [35].

The gut microbiome shares a commensal relationship with the host by deriving nutrients from the gut cells and in turn performing several functions for the host’s physiology [6]. Importantly, besides metabolizing several large macronutrients, the gut microbiome shapes both the innate and adaptive immune systems of the host [41]. In addition, gut microbes have been found to control brain development and function and thus to influence the host’s behavior [61, 90].

## ASD and gut dysbiosis

Extensive studies conducted in the last few years have shown the important role that the gut microbiome has in influencing the development of the nervous system. In doing this, the gut microbiome is in the unique position of modulating behavior, not only in normal conditions but also when neurological disorders, including ASD, arise [23, 61, 90]. Consequently, several studies have shown that gastrointestinal disease symptoms such as diarrhea and constipation are commonly observed in ASD patients, many of which also show abnormal behavioral patterns such as aggression, anxiety, and tendency to self-injury [15, 94]. Furthermore, an important correlation between changes in gut bacteria composition and personality was recently reported. Individuals who were less careful and diligent tended to have lower abundance of *Proteobacteria* [43]. Moreover, ASD patients have significantly higher intestinal permeability which causes leakage of lymphocytes and pro-inflammatory cytokines into the circulatory system. Those inflammatory molecules eventually reach the brain and cause immune activation there [3, 4]. As gut dysbiosis is responsible for the increased permeability of the intestine epithelial cells, this evidence supports the idea that there is an important effect of gut dysbiosis on immune dysregulation and possibly on ASD [72].

Along the gut microflora of the ASD patient, the maternal gut microbiome has also been found to be influential in the development of ASD in the offspring. Animal studies using the maternal immune activation (MIA) mouse model have shown that administration of the antibiotic vancomycin to MIA-subjected pregnant dams prevented the deleterious effects of MIA on the offspring. Specifically, offspring from antibiotic-treated female mice did not show the brain structural abnormalities or ASD-like behavioral phenotype observed in the offspring of untreated MIA-exposed mice [14]. Further evidence came from a recent study showing that orally administering a commercially available probiotic formula to pregnant dams prior to MIA induction prevented ASD-like behavior when compared to the offspring from MIA females ( [95]). Taken together, these recent findings point towards a strong relation between ASD and either their own, and/or their maternal gut microbial dysbiosis.

## ASD and immune dysregulation

Substantial evidence from both human and animal studies has linked ASD with the patient’s own immune system imbalance. For instance, several studies have found abnormalities in both innate and adaptive immunity in ASD patients, including elevated levels of Th-1 and Th-2 cytokines [19, 36]. Such imbalance in inflammatory pathways could contribute to a potentially dysfunctional blood-brain barrier and consequent compromised brain function [89]. Multiple studies comparing serum immunoglobulins as well as pro-inflammatory cytokine levels have shown that ASD patients have increased levels of IL-1β, IL-6, IL-8, and IL-12p40 [5], together with increased tumor necrosis factor alpha (TNF-α) and interferon gamma (IFN-γ) levels [19]. These higher cytokine levels were associated with communication deficits and aberrant behaviors, in fact higher TNF-α plasma levels were correlated with more severe ASD symptoms. Other studies have demonstrated that increased levels of IL-17A in children affected with ASD positively correlate with the severity of ASD symptoms [2]. Taken together, the increase in proinflammatory cytokines (such as IL-6, IL-12, and IFN-ɣ), and the decrease in anti-inflammatory cytokines (such as IL-10 and TGF-β1) suggests a possible state of overactive immunity in ASD patients [33]. A recent meta-analysis of 38 studies including 2487 participants (1393 patients with ASD and 1094 control subjects) provided evidence for a significantly higher concentration of pro-inflammatory cytokines IFN-γ, IL-1β, IL-6, and TNF-α in autistic patents compared with age-matched control subjects. Including meta-regression analyses also indicated significant interaction of latitude, age, and gender [77]. Furthermore, not only the levels of pro-inflammatory molecules were found to be altered in ASD patients, but also properties of cells isolated from ASD patients retained altered responses. In an in vitro study comparing the cytokine response to stimulation by toll-like receptor (TLR) ligands showed a striking pro-inflammatory response in cultured monocytes isolated from ASD patients compared with those from age-matched controls ([27]).

Another line of evidence for immune system compromise in ASD is the finding of maternally originated antibodies against neuronal and glial proteins (also termed anti-brain antibodies) that have been documented in ASD patients [18, 83, 84]. The presence of such anti-brain antibodies in ASD patients was associated with cognitive and behavioral dysfunctions [68]. These maternal antibodies are clearly different from the autoantibodies that have also been reported in children with ASD ([26, 32].). It is important to note that no direct evidence has been provided that antibodies from an affected mother have been transmitted to a fetus (through the placenta) and that these have caused ASD [33]. Findings from a combination of animal and human studies have shown an association between maternal antibodies and ASD in the progeny [11]. For example, exposing pregnant Rhesus monkeys to IgG antibodies from human mothers of ASD children resulted in atypical stereotyped behaviors compared to controls injected with IgG from mothers of typically developing children [53]. Similarly, injecting serum antibodies from a mother of children with ASD into pregnant mice resulted in altered behavior and sociability in the offspring compared with offspring of mice injected with serum from mothers of healthy children [22, 82]. Recent studies aiming at discovering how these maternal antibodies function have shown clear differences in their binding properties when comparing maternal antibodies from samples from ASD and typically developing children [25]. More specifically, these studies have found that mothers of children with ASD have autoantibodies that are able to bind to a more diverse set of peptides, with specific combinations of peptides not observed in antibodies from mothers of typically developing children.

Parental and familial dysfunctional immune system have also been previously correlated with ASD. For instance, approximately 37% of surveyed ASD children reported maternal or first-degree familial autoimmune disorder whereas only 6% of the control group showed this condition [16]. Further studies showed that families with autoimmune disorders such as type-1 diabetes, RA, autoimmune thyroid diseases, and systemic lupus erythematosus (SLE) have a higher chance of having children diagnosed with ASD [58, 87]. Some studies have suggested that the presence of anti-brain antibodies in the pregnant mother causes developmental alterations in the child’s brain [12]. This idea received experimental support in a mouse model where the administration of anti-brain antibodies to pregnant dams resulted in ASD-like behavioral abnormalities in their offspring [82]. For these reasons, in recent years, there has been an increase in ASD research devoted to use MIA as animal model. Thus, it has been reported that injection of poly(I:C) synthetic virus in pregnant dams at their embryonic day 12.5 (E12.5) increases the maternal serum IL-6 which in turn causes ASD-like phenotype in the offspring [85]. More recently, another study showed that MIA results in the elevation in the maternal serum IL-17 level which also causes ASD-like behavior in the offspring. This study reported that in fact maternal IL-17 level was the causal factor for the offspring’s ASD as the administration of an IL-17 neutralizing antibody prevented the occurrence of ASD. Additionally, this study also found IL-17 receptors in the fetal brain [17]. These findings shine light on the possibility that maternal IL-17 could bind to these specific receptors in the fetal brain during the developmental phase of the nervous system, then initiating a signaling cascade (s) that may lead to structural and behavioral abnormalities [98].

## High salt diet, gut dysbiosis and immune dysregulation

Excessive salt intake is a major worldwide public health issue. First, it is well known that consumption of elevated dietary salt can lead to several cardiovascular diseases. According to the WHO, the top two major causes of death globally are ischemic heart disease and stroke, which can be caused by consuming too much salt [63]. Second, salt consumption indeed exceeds the recommended level almost everywhere where there is a record of it, with very little difference between countries [69]. On average, salt consumption currently reaches 9–12 g per day whereas the WHO recommends a daily salt intake of less than 5 g per day (2014). Indeed, approximately 2.5 million of deaths worldwide could be prevented each year by reducing the daily salt intake ([13]; 2014). Third, most of the excess of salt in food is added during the manufacturing process. In the USA, more than 70% of daily sodium, a major component of salt, comes from processed foods, 14% naturally occurs in the food ingredients and only 5% is being added while cooking [37]. It is also alarming that, according to the US CDC, the foods that contain the highest levels of salt are the foods that we consume more often, such as a variety of highly processed items (e.g., canned foods, pizza, cured meats, popcorns, chips).

**Fig. 1:**
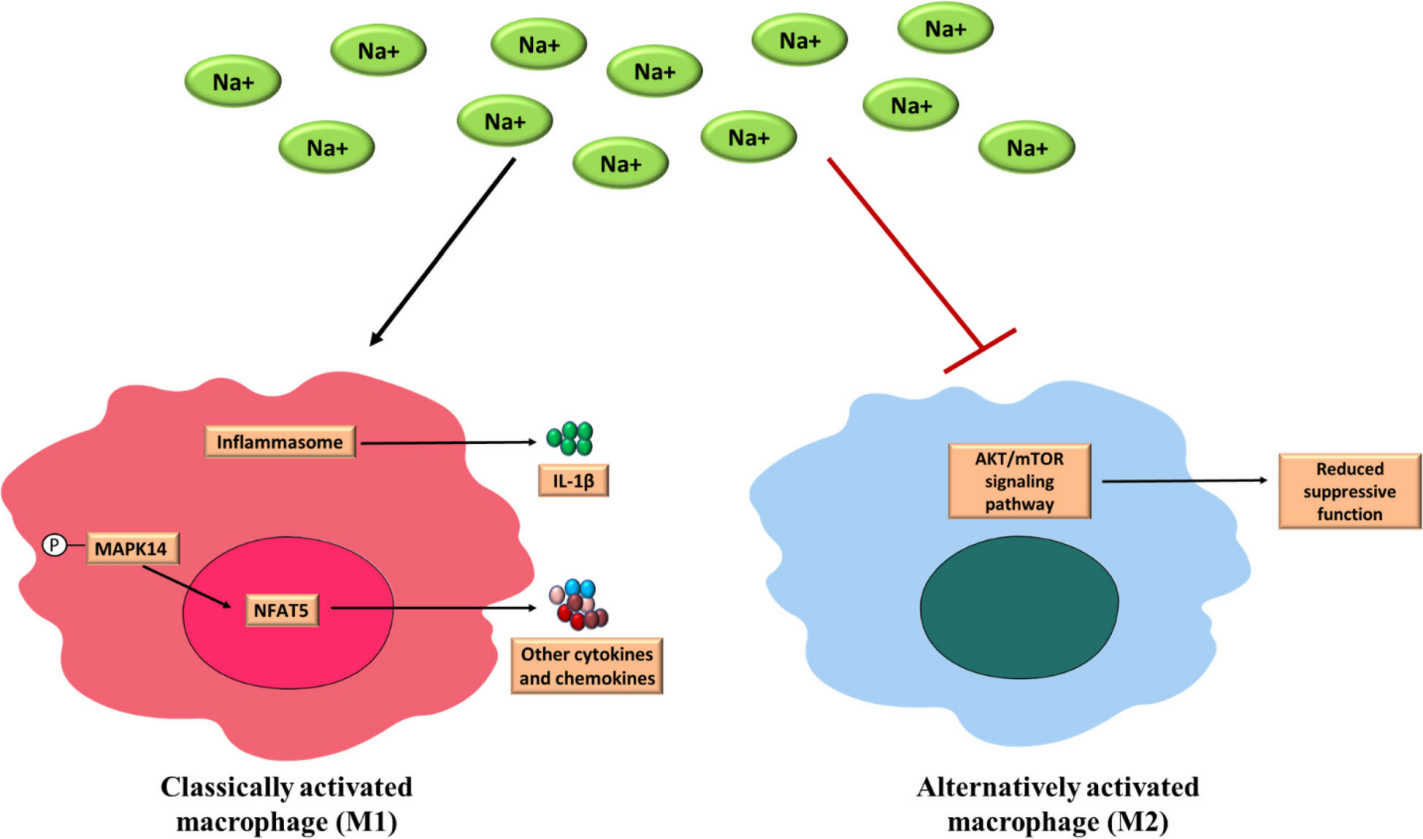
Effect of high salt on innate immunity. A high salt condition increases pro-inflammatory macrophages (M1) and activates inflammasomes that lead to the production of interleukin (IL)-1β. It also activates the MAPK pathway and increases the production of several chemokines and cytokines. High salt can also reduce the activity of alternative (cell repairing), non-inflammatory macrophages M2 (which produce regulatory cytokines such as IL-10) by dysregulating their AKT/mTOR signaling pathway

The cellular effects of excessive salt on innate immunity are portrayed in Fig. 1. The mononuclear phagocyte system (MPS) cells of the innate immune system, such as, macrophages or dendritic cells of the skin interstitium respond to extracellular hypertonicity caused by excessive salt. Macrophages secrete vascular endothelial growth factor-C in response to the excessive presence of sodium (Na^+^) [51]. Further, excessive salt can also dysregulate how macrophages are activated, thus disrupting the immune system homeostasis [102]. For example, hypertonic conditions created by higher salt concentration increase pro-inflammatory macrophages (M1) and activate inflammasomes that lead to the production of IL-1β [40, 60]. High salt concentration also activates the mitogen-activated protein kinase (MAPK) pathway in the M1 macrophages and increases the production of several chemokines and cytokines [102]. Moreover, high salt can also reduce the activity of the cell-repairing non-inflammatory M2 macrophages. This alternative pathway produces anti-inflammatory cytokines such as IL-10 by dysregulating AKT/mTOR signaling pathway [9].

**Fig. 2:**
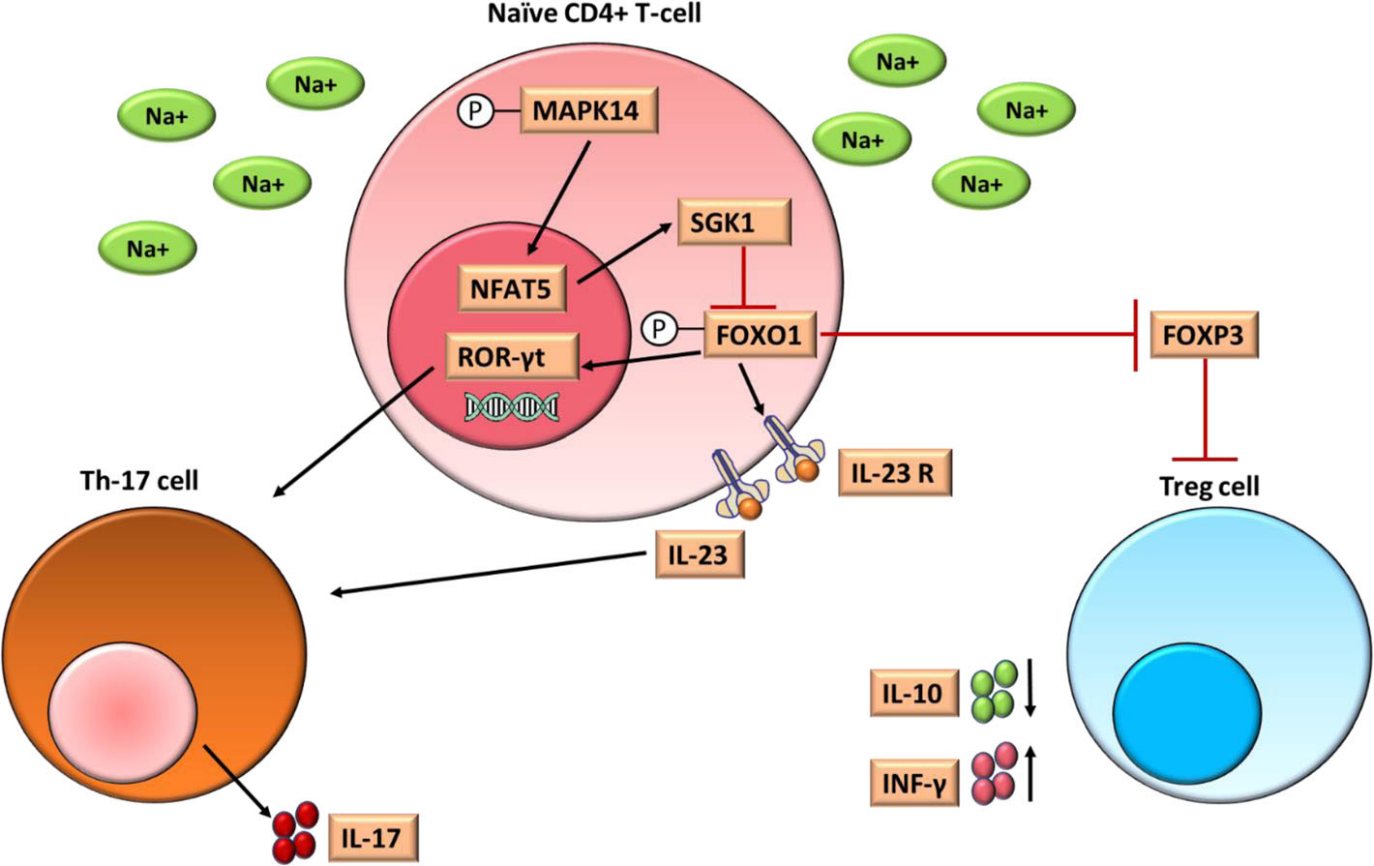
Effects of high salt on the adaptive immune system. High concentration of salt increases the phosphorylation of the intracellular signaling molecule MAPK14 in the naïve CD4+ T cell, which also activates the transcription factor NFAT5. High salt also upregulates serum glucocorticoid-regulated kinase 1 (SGK1). Activated SGK1 phosphorylates forkhead box protein O-1 (FOXO1). Phosphorylated FOXO1 cannot bind to the IL-23 receptor (IL-23R) promoter which allows increased transcription of IL-23R as well as increased production of IL-23. Increased IL-23 level is a major stimulus for Th-17 cell differentiation. The phosphorylated FOXO1 protein also induces the expression of retinoic acid-receptor-related orphan receptor γt (RORγt), a transcription factor of Th17 cell differentiation Additionally, activated SGK1 and deactivated FOXO1 decrease the expression of FOXP3 which eventually reduces the differentiation of Treg cells. High salt also increases the INF-γ production from Treg cells, which decreases its suppressive activity

High salt intake can also influence the adaptive immune system (Fig. 2). First, the increased level of IL-1β has the potential to increase the differentiation of Th-17 cells [60]. Moreover, several in vitro and in vivo studies have reported that high salt can induce the differentiation of Th17 cells as well as the production of IL-17 from naïve CD4+ T cells by activating several signaling pathways [9, 45, 47]. Growing Th17 cells in conditions mimicking the salt concentration found in the interstitium of a high salt fed animal (i.e., in medium containing < 80 mM sodium chloride) demonstrated that high salt increases the phosphorylation of the intracellular signaling molecule MAPK14 and the transcription factor NFAT5 (nuclear factor of activated T cells 5). Furthermore, high salt also upregulated the serum glucocorticoid-regulated kinase 1 (SGK1) [9, 45]. Activated SGK1 phosphorylates forkhead box protein O1 (FOXO1) [99]. In normal conditions, unphosphorylated FOXO1 proteins bind to the IL-23 receptor (IL-23R) promoter and suppresses its transcription. Phosphorylated FOXO1 however cannot bind to the IL-23R promoter, therefore allowing increased IL-23R transcription and IL-23 production, which is a strong stimulus for Th17 cell differentiation [45]. The phosphorylated FOXO1 protein can also induce the expression of a transcription factor of Th17 cell differentiation named retinoic acid-receptor-related orphan receptor γt (RORγt) [9, 45, 47]. Along with this, feeding high salt diet to mice subjected to experimental autoimmune encephalomyelitis (EAE), a mouse model of Multiple Sclerosis (MS), showed significant worsening of their condition [45, 99]. These findings agree with the effect of high salt diet on Th-17 production as MS is strongly associated with increased Th-17 differentiation [24]. Further, increased salt intake in MS patients was associated with exacerbation of the disease progression [29].

Similarly, high salt consumption can affect regulatory T (Treg) cells. Treg cells downregulate the effector T cell proliferation and induction [8]. Though Treg cells function as suppressors, they are also involved in the production of pro-inflammatory cytokines such as IFN-γ and IL-17. High salt consumption has been shown to induce a 6-fold increase in IFN-γ secretion from Treg cells; this overproduction of IFN-γ in turn decreases the suppressive function of Treg cells [21]. Further, in vitro and in vivo data showed that activated SGK1 caused by high salt consumption can regulate CD4+CD25hiCD127loFoxp3+Treg cells which results in functional loss of modulatory ability [39]. Moreover, the excessive differentiation of Th17 cells caused by high salt is associated with cognitive dysfunction and memory impairment by causing a reduction in cerebral blood flow due to reduced endothelial NO production [28].

Recent studies have indicated that the immune dysregulation caused by high salt diet is mediated by gut dysbiosis [60, 78]. For instance, mice fed with excessive amounts of salt (4–8% for 4–8 weeks) showed significant changes in their gut microbial composition [56]. This high salt regimen specifically reduced several species of genera *Lactobacillus, Oscillibacter, Clostridium* XIVa, and *Rothia*, while increasing *Parasutterella* [56, 96]. Interestingly, this study also showed that the gut dysbiosis in the high salt diet fed mice resulted in dysregulation of Th17 cells, which are responsible for the over production of IL-17. In addition, the abnormal inflammation observed in the high salt diet fed mice was reversed by the administration of two species of *Lactobacillus*, e.g., *L. murinus* and *L. reuteri* [96].

## Maternal elevated salt consumption and ASD in the offspring

**Fig. 3:**
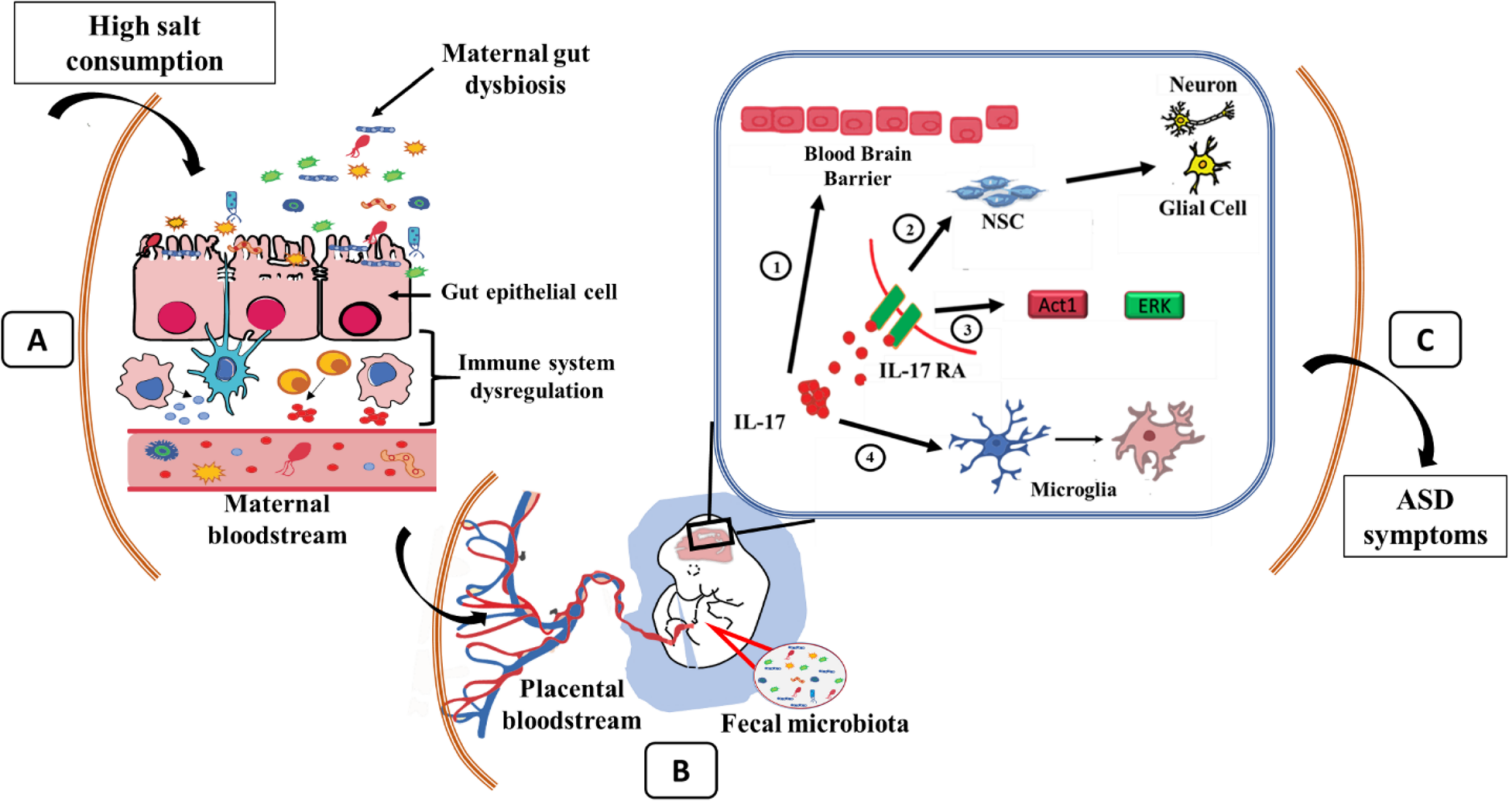
Maternal high salt diet and offspring ASD. Chronic consumption of high salt causes microbial dysbiosis in the maternal gut (A), which can then result in maternal immune system dysregulation: (**a**) increased differentiation of Th-17 cells and concomitant increased IL-17, (**b**) increased M1 macrophage activation with related cytokines and chemokines release, and (**c**) decreased Treg cell differentiation and regulatory M2 macrophage activation. The altered maternal gut microbiota and increased IL-17 travel via placental circulation to the fetus (B). In the fetal intestine, the gut microbiota and increasing IL-17 serum levels could cause a similar immune dysregulation as in the maternal side. When circulating IL-17 reaches the fetal brain and causes the activation of IL-17 receptor A (IL-17RA), several mechanisms can be triggered: (1) IL-17 could hamper the development of the fetal blood brain barrier (BBB), (2) reduction of the proliferation of neuronal stem cells (NSC) which in turn decreases neuro and gliogenesis, (3) dysregulation of the Act1 signaling pathway which alters the expression of ERK protein, and (4) IL-17 can also over-activate microglia. The described changes contribute to the ASD-like phenotype observed after birth (C)

Maternal diet and gut microbiome are factors that have been received considerable attention from ASD researchers. However, among several dietary components that have been studied in detail, the effect of maternal high salt diet has not received comparable consideration. A review of recent literature suggests a possible causal link between maternal salt consumption and offspring ASD. On the one hand, several lines of evidence have shown that elevated salt intake can alter both the innate and adaptive immunity via maternal gut dysbiosis. Such abnormal gut microbiome result in an increased differentiation of Th-17 cells, with concomitant hyper production of their effector IL-17 [45]. The higher differentiation of Th-17 cell as well as the presence of high concentrations of IL-17 in the blood play a major role in the progression of several autoimmune disorders such as RA, SLE, MS, and autoimmune thyroid disorder [31, 38, 50, 75]. Further, several recent studies have shown that the maternal gut microbiome present during pregnancy and breastfeeding can be transferred to the fetus and newborn via placental blood circulation and breast milk [35, 93]. In contrast, administration of an IL-17 specific antibody and a probiotic supplement to MIA pregnant mice showed the attenuation of ASD in the offspring which establishes a possible causal relationship between maternal gut dysbiosis and immune dysregulation resulting in offspring ASD [17, 95]. Thus, we hypothesize that there might be a potential causal relationship between maternal high salt consumption and offspring ASD (Fig. 3). Specifically, chronic consumption of high dietary salt causes gut dysbiosis and dysregulation of the Th17 axis [56, 60, 96]. Both conditions would continue during a possible pregnancy, with the concomitant increase in Th17 cell differentiation as well as higher level of serum IL-17 in the pregnant female that could be transferred through the placental blood to the fetus. When IL-17 reaches the developing fetal brain, it could affect the development of the fetal blood-brain barrier (BBB) as it has been reported in a mouse model that the formation of a fully functional BBB is completed at embryonic day 15.5 (E15.5) [7]. The development of a dysfunctional BBB may increase its permeability and thus cause the infiltration of effector molecules (including several cytokines) into the fetal brain. Furthermore, as the altered gut microbiome is shared from the mother to the fetal intestine, a higher number of immune cells are differentiated there and can produce excessive pro-inflammatory cytokines [41, 93]. The altered gut microbial composition can also increase the permeability of fetal intestinal cells and cause the infiltration of those cytokines to the blood stream [72]. Additionally, IL-17 from the placenta can potentially act on fetal brain cells expressing IL-17 receptors (IL-17RA) [17]. In normal conditions, IL-17RAs have been shown to have a very low expression [98]; however, it remains to be determined whether in conditions of gut dysbiosis-induced fetal brain immune activation, IL-17RA expression is upregulated. Nevertheless, when IL-17 binds and activates fetal IL-17RAs, this could affect the development of the fetal brain in several possible ways. Firstly, the activated IL-17RA may cause neuronal death as well as inhibition of neural stem cell (NSC) proliferation. Secondly, it may overly activate microglia which can cause NSC apoptosis, decreased neuronal differentiation, and increased glial cell differentiation. Furthermore, the activation of IL-17RA can activate Act1, the signaling molecule downstream from IL-17RA. The activated Act1 can then activate several other signal transduction pathways including the extracellular signal-regulated kinase (ERK) pathway. The ERK pathway is associated with several essential function in the nervous system including neuronal differentiation, synaptic plasticity, and cognition [20, 34, 65]. Interestingly, MAPK/ERK signaling pathway is hyperactivated in ASD patients [92]. Further studies showed the increased level of phosphorylated MAPK and phosphorylated ERK in the prefrontal cortex area of ASD mouse model brain [30]. Additionally, inhibition of the phosphorylation of ERK protein during the developmental period of mice fetal brain was found to rescue the behavioral deficits [71]. Thus, IL-17 RA-mediated Act1 activation may cause the increased phosphorylation of ERK in the fetal brain hindering its development as well as behavior.

Overall, the existing evidence supports the hypothesis that there is a possible association between maternal elevated salt consumption and offspring ASD, although more research is needed to validate this. The idea is especially concerning considering the documented excessive consumption of salt and the increased prevalence of ASD.

## Maternal diet and epigenetic effects

Epigenetic modifications refer to molecular factors that influence genetic activity without changing our inherited primary DNA sequence. These epigenetic marks change the local chromatin environment and therefore affect DNA accessibility, regulating gene transcription and a wide range of processes associated with the DNA [101]. Further, a multitude of studies have suggested that epigenetic marks are also sensitive to environmental exposure. In fact, environmental factors such as nutrients, contaminants, toxins, and others have been shown to impact the levels and turnover of epigenetic modifications and therefore can alter gene expression patterns possibly resulting in disease [57]. One of the most well-known examples of epigenetic regulation is DNA methylation, which generally correlates with transcriptional silencing when located in a gene promoter [42]. Interestingly, DNA methylation has been implicated in the pathophysiology of ASD [46]. Then, if we consider high salt diet as an environmental factor, it is relevant to think about potential epigenetic mechanisms contributing to cause ASD in the offspring of mothers (and/or fathers) that have consumed too much salt. In this context, elevated salt has been shown to alter the DNA methylation levels of genes related to aldosterone biosynthesis [59], while also influencing the activity of histone-modifying enzymes associated with salt-induced changes in blood pressure, such as lysine-specific demethylase 1 (LSD-1) [97]. However, a clear connection between epigenetic mechanisms induced by high salt diet and ASD is yet to be observed. Further studies are needed to improve of our understanding of the involved mechanisms when these different pathways crosstalk. Nevertheless, there is indirect evidence of putative intersection between high salt, epigenetics, and ASD, from a wealth of literature indicating substantial epigenetic changes in the immune system [54], in particular changes related to autoimmune disorders. Remarkably, as discussed earlier, there are many studies showing a clear association between autoimmunity disorders in parents and ASD in their children.

## Conclusions and future studies

With the growing number of patients diagnosed with ASD, the urge to find causal factors and mechanisms involved is also rising. While studies have shown clear correlations between dietary habits and gut dysbiosis with a variety of illnesses related to immune dysregulation (including brain disorders), whether maternal (and/or paternal) dysfunctional immune system induced by elevated salt intake (and mediated by gut dysbiosis) has any effect on children’s brain development has not been explored in detail. Extensive research is needed to first identify a causal association, and second, to uncover the mechanisms involved in linking excessive salt, gut dysbiosis/immune compromise, and ASD in the offspring. Moreover, most of the previous research involving salt intake and immune dysregulation has been done using a mouse model and using an amount of salt almost 16-fold higher than their daily requirement. As the sodium metabolism in mice and humans is different, further research is necessary to elucidate the lower and upper limits of consumed salt that can cause immune dysregulation in humans. If our hypothesis (i.e., there is a causal relation between maternal salt consumption and children’s ASD) is experimentally validated, several therapeutic options will be available for the treatment and possible prevention of the disorder. Additionally, dietary salt could be considered as a risk factor not only directly for the consumer’s health (i.e., adults that will eventually reproduce), but also for their progeny.

A better understanding of environmental factors (such as dietary choices) that increase the probability of ASD, and other neurological disorders, could have a tremendous impact in determining public health policies. In the future, implementing such interventions, specifically during pregnancy, could reduce the burden of inadequate access to resources and information regarding dietary habits.

An important research avenue to explore is to continue developing epigenetic studies. The directions to follow are diverse but one of the most essential areas is to provide the evidence of causality between high salt diet, gut dysbiosis, and compromised immune system, and the development of ASD in the offspring. So far, multiple associations have been found amongst some of these factors, but there is no agreement yet on a unified theory. Further epigenetic studies, nonetheless, could help uncovering biomarkers for a more accurate prediction of risk of ASD prior to diagnosis, as well as severity and penetrance.

## Data Availability

N/A

## Abbreviations

ASD: Autism spectrum disorder
EAE: Experimental autoimmune encephalomyelitis
ERK: Extracellular signal-regulated kinase
FOXO1: Forkhead box protein O1
IFN-γ: Interferon gamma
IL: Interleukin
IL-17RA: IL-17 receptors
MAPK: Mitogen-activated protein kinase
MIA: Maternal immune activation
MS: Multiple sclerosis
mTOR: Mammalian target of rapamycin
NFAT5: Nuclear factor of activated T cells 5
NO: Nitric oxide
NSC: Neural stem cell
RA: Rheumatoid arthritis
RORγt: Retinoic acid-receptor-related orphan receptor γt
SGK1: Serum glucocorticoid-regulated kinase 1
SLE: Systemic lupus erythematosus
Th: T helper cells
TNF-α: Tumor Necrosis Factor alpha
Treg: Regulatory T cells
WHO: World Health Organization
